# Analysis of Legislative and Regulatory Frameworks Governing Community Pharmacy in Bulgaria and North Macedonia

**DOI:** 10.3390/pharmacy13040108

**Published:** 2025-08-08

**Authors:** Anna Todorova, Mariya Ivanova, Magdalena Pesheva, Dijana Miceva, Bistra Angelovska

**Affiliations:** 1 Faculty of Pharmacy, Medical University of Varna, 9000 Varna, Bulgaria; anna.todorova@mu-varna.bg (A.T.); magdalena.pesheva@mu-varna.bg (M.P.); 2 Faculty of Medical Sciences, Goce Delcev University, Krste Misirkov Str., No. 10-A, P.O. Box 201, 2000 Stip, North Macedonia; dijana.miceva@ugd.edu.mk (D.M.); bistra.angelovska@ugd.edu.mk (B.A.)

**Keywords:** community pharmacies, pharmacists, pharmaceutical legislation, EU, Bulgaria, North Macedonia

## Abstract

The common border between Bulgaria and North Macedonia, alongside the regulatory requirements stemming from Bulgaria’s membership in the European Union, provide grounds for comparing the legislative environment in both countries. This article presents a comparative case study of the regulatory frameworks governing community pharmacies in Bulgaria and North Macedonia. The aim of this study is to examine the specific features of current legislation related to the operation of community pharmacies, and to identify similarities, differences, and gaps in the organizational structure of pharmacy services, the population’s access to pharmaceutical care, and the qualification requirements for personnel working in community pharmacies. Bulgaria has been a member of the European Union since 2007, while the Republic of North Macedonia has had official EU candidate status since 2005. This provides a basis for comparing the regulatory frameworks of an EU and a non-EU system within the same regional context. In both countries, the overall pharmacy-to-population ratio exceeds the European average (3.3 pharmacies per 10,000 inhabitants), indicating sufficient availability. However, pharmacies are predominantly concentrated in major urban areas. In Bulgaria, challenges remain in ensuring access to pharmaceutical services in smaller and rural settlements, while in North Macedonia, the provision of such services is better ensured. The findings of this case study may be particularly relevant for countries undergoing health system reforms or EU harmonization processes.

## 1. Introduction

Bulgaria is a member of the European Union, and its legislative framework regulating the trade in medicinal products is fully aligned with European legislation. One of the fundamental roles of the Union is to ensure the free movement of goods and/or services, which includes pharmaceutical market products. In order to harmonize the requirements for medicinal products with the aim of ensuring their quality, safety, and efficacy, a unified pharmaceutical regulatory system has been established at the European level, minimizing national discrepancies. These common regulatory standards also contribute to improving public access to medicinal products across all regions. To safeguard public health within the European Union, the importation of medicinal products is subject to specific regulatory requirements, including import authorization, marketing authorization, rules for labelling and packaging, batch control, and a pharmacovigilance system [1]. Pharmaceutical regulation in the EU has evolved from a nationally oriented system to an increasingly international one [2]. The establishment of the Single Market is supported and protected by a comprehensive set of EU-level regulatory measures, including numerous regulations and directives.

The European Medicines Agency (EMA) was established in 1995 to harmonize pharmaceutical regulation across the European Union. Its mandate includes safeguarding public health and promoting the rational use of medicinal products in both human and veterinary medicine [3]. The European Directorate for the Quality of Medicines and Healthcare (EDQM) was also established to oversee the quality of medicines by developing standards for the analysis of active substances and excipients [4]. Additionally, it monitors the quality of pharmaceutical practice and pharmaceutical care.

Upon Bulgaria’s accession to the European Union, its national legislation was adapted to the EU pharmaceutical regulatory framework. In 2007, the Medicinal Products in Human Medicine Act (MPHMA) was adopted, incorporating EU requirements into national law [5].

The MPHMA is primarily based on Directive 2001/83/EC [2], which constitutes the Community Code relating to medicinal products for human use.

The Republic of North Macedonia remains a candidate country for EU membership. In September 2004, the Macedonian government adopted a National Strategy for European Integration [6]. On 17 December 2005, the European Council officially granted North Macedonia candidate status, following a review and positive recommendation from the European Commission [7]. At present, national legislation governing the pharmaceutical sector in North Macedonia does not mandate full alignment with EU law. However, steps have been taken to harmonize pharmaceutical regulation with EU standards. In 2007, the Law on Medicines and Medical Devices was revised, and a number of implementing regulations were adopted [8].

In both countries—Bulgaria and the Republic of North Macedonia—the functions of evaluation and oversight of medicinal products and medical devices are carried out by specialized national regulatory bodies under the Ministries of Health.

In Bulgaria, this body is the Bulgarian Drug Agency (BDA). It is a specialized authority overseeing the quality, safety, and efficacy of medicines. The BDA is a legal entity financed from the state budget, headquartered in Sofia, and it operates under the authority of the Minister of Health. In the Republic of North Macedonia, the equivalent body is the Macedonian Agency for Medicines and Medical Devices (MALMED). It is also an independent legal entity funded by the state, with its headquarters in Skopje.

This divergence presents an opportunity to explore how differences in legislation may influence the organization of pharmacy practice and the roles of pharmacists in the two countries. Given these regulatory and professional differences, it is relevant to conduct a comparison of pharmaceutical legislation and pharmacy practice in Bulgaria and North Macedonia. The findings would be significant for public health policy, particularly in supporting access to pharmaceutical care and guiding alignment with EU standards.

Purpose: The aim of this study is to examine the specific features of current legislation related to the operation of community pharmacies, and to identify similarities, differences, and gaps in the organizational structure of pharmacy services, the population’s access to pharmaceutical care, and the qualification requirements for personnel working in community pharmacies.

## 2. Regulation and Oversight of the Retail Trade of Medicinal Products

Historically, following the democratic changes in Bulgaria, the first pharmaceutical act—the Medicinal Preparations and Pharmacies in Human Medicine Act—was adopted in 1995, marking the beginning of the country’s alignment process with European pharmaceutical legislation. This act laid the foundation for pharmaceutical regulation through the adoption of secondary legislation (regulations and ordinances) covering key areas such as manufacturing, registration, clinical trials, use, import, export, wholesale, and retail trade of medicinal products for human use. The aim was to ensure the quality, efficacy, and safety of these products [9].

In 2000, the act was renamed the Medications and Pharmacies in Human Medicine Act (MPHMA), and in accordance with its provisions, the Bulgarian Drug Agency (BDA) was established. The Agency serves as the national competent authority responsible for the supervision of the quality, efficacy, and safety of medicinal products in Bulgaria.

From 1996 to 2005, the MPHMA was amended and supplemented multiple times, which complicated its enforcement [10]. Despite numerous amendments, the act did not fully align with European directives on medicinal products This necessitated the development of a new law aimed at achieving full harmonization of Bulgarian pharmaceutical legislation with European Union law and establishing conditions that ensure the use of medicinal products that meet the requirements for quality, safety, and efficacy.

In 2007, the Medicinal Products in Human Medicine Act (MPHMA) came into effect. Its aim was to implement European legislation and it remains in force today. According to the MPHMA, the retail trade of medicinal products can only be carried out in pharmacies and drugstores. Individuals or legal entities registered under the Commercial Code or the legislation of an EU member state have the right to open a pharmacy or drugstore. The structure, procedures, and organization of pharmacy operations, as well as the nomenclature of medicinal products, are determined by a regulation issued by the Minister of Health [11]. A separate regulation addresses the conditions and procedures for organizing operations in drugstores [12].

The Republic of North Macedonia emerged as an independent state following the declaration of its independence in 1991. Since then, the country’s pharmaceutical sector has undergone numerous reforms.

Retail trade in medicines in the Republic of North Macedonia is regulated by two main laws: the Law on Medicines and Medical Devices (2007) and the Law on Health Care [8,13]. The operation of pharmacies is further governed by the Rulebook regarding premises, equipment, and professional staff for establishing, starting work, and performing health activities in healthcare institutions [14]. In the Republic of North Macedonia, the retail sale of medicines is carried out exclusively in pharmacies, which are established in accordance with the Law on Medicinal Products and Medical Devices; the Law on Health Care; the Rulebook on the required space, equipment, and qualified personnel for establishing, starting, and performing healthcare activities in health institutions; the regulation on the network of health institutions; the Law on Health Insurance; and other legal and sublegal acts.

In both countries, Good Pharmacy Practice (GPP) Guidelines have been adopted as a system of standards aimed at ensuring the provision of high-quality pharmaceutical services, fostering a professional attitude of community pharmacists toward patients, and promoting self-assessment and professional oversight. The core principles of GPP ensure pharmacists’ involvement in the rational and safe use of medicines. These guidelines were developed based on the recommendations of the World Health Organization (WHO) and the International Pharmaceutical Federation (FIP) for the creation of national GPP frameworks.

In Bulgaria, the GPP guidelines were developed by the Quality Committee of the Bulgarian Pharmaceutical Union (BPhU), based on the Professional Organization of Master Pharmacists Act and in accordance with the recommendations of the WHO and the FIP. They were officially adopted by the Governing Board of the BPhU and endorsed by the Minister of Health in 2009 [15]. During the same year, North Macedonia published its Good Pharmacy Practice Rules in the official State Gazette and formally implemented them [15,16].

In both countries, medicines agencies have regulatory control and oversight, with the primary objective of ensuring that only high-quality, safe, and effective medicinal products reach patients through supervision of the entire supply chain from manufacturing to retail distribution.

## 3. Results

### 3.1. Infrastructure and Access to Pharmacies and Pharmaceutical Services

In both Bulgaria and North Macedonia, there are two main types of pharmacies: community pharmacies, which serve the general population, and hospital pharmacies, which serve the needs of healthcare facilities.

Currently, in both countries, community pharmacies are privately owned. Authorization to establish a pharmacy is granted by the respective national medicines agency and is issued for an indefinite duration.

In Bulgaria, the emergence of private pharmacies began during the country’s post-1990 transition from a centrally planned economy to a democratic system based on market principles and private property. This shift enabled the liberalization of the pharmaceutical sector and the proliferation of privately owned pharmacies.

Similarly, North Macedonia underwent a transition from a planned to a market economy, which significantly affected the organization and financing of the healthcare sector. The first private pharmacy in the country was established in 1992. At that time, municipal pharmacies remained under state ownership and were funded by the Health Insurance Fund of the Republic of North Macedonia (HIF).

Until the year 2000, private pharmacies were not eligible to enter into contractual agreements with the HIF, and publicly reimbursed medicines were dispensed exclusively through state-owned pharmacies. Nonetheless, due to persistent shortages of essential medicines in public outlets, the number of private pharmacies grew steadily, despite the fact that patients were required to pay out of pocket for medications included in the reimbursement list.

The situation changed substantially once private pharmacies were authorized to dispense reimbursed pharmaceuticals, leading to a marked increase in their number. Following the privatization of state-owned pharmacies in 2007, all community pharmacies in North Macedonia have since been under private ownership [17]. Mobile pharmacies are expected to be permitted in North Macedonia to supply medicines in settlements with populations of up to 1000 residents. The establishment and operation of mobile pharmacies are expected to be permitted, contingent upon legislative justification that ensures regulatory compliance and safeguards public health. These mobile units will function as extensions of healthcare institutions, staffed by professionals with a pharmaceutical education (either tertiary or secondary), and will be supervised at least once per week by a licensed pharmacist holding a university degree.

A comparative overview of the legal requirements related to ownership and types of pharmacies in the two countries is presented in Table 1.

In Bulgaria, according to the MPHMA, the owner of a pharmacy can be any natural or legal person registered as a trader, who has a labor or management contract with a qualified master pharmacist with at least one year of professional experience. In North Macedonia, the owner of a pharmacy can be any legal entity that has no conflict of interest with the institutions overseeing pharmacies and wholesale medicine distributors [8]. Pharmacies are licensed and regulated by the Ministry of Health through the National Medicines Agency [18].

The two countries have different legislative restrictions on pharmacy ownership. In Bulgaria, each owner is allowed to own up to four pharmacies. In North Macedonia, there are no restrictions on horizontal integration and the number of pharmacies that a physical or legal person may own.

Vertical integration in both countries is subject to legislative regulation. In Bulgaria, a master pharmacist or a pharmacy assistant who is a holder of the retail pharmacy permission cannot be employed under a contract with a sole trader or a company whose business includes the production, import, wholesale, or retail trade in medicinal products. If the master pharmacist is the manager of only one pharmacy, they must work there full time and have no right to work at any other location. In North Macedonia, individuals holding positions in the Medicines Agency, the Ministry of Health, or the Health Insurance Fund, or those with a relative working in any of these institutions, are prohibited from opening a pharmacy. Despite the legal restriction on vertical affiliation (the owner of a wholesale trader or industrial enterprise and related individuals cannot own pharmacies), there are pharmacy chains, the largest of which are linked to wholesalers of pharmaceutical products, as well as manufacturers.

Since 2015, there has been a requirement for a distance of more than 100 m between pharmacies in towns with up to 4500 inhabitants. In towns with a population of more than 30,000—and, under specific conditions, in towns with up to 30,000 residents—non-prescription (over-the-counter) medicinal products may be sold in grocery stores and gas stations. In towns with populations above 30,000, pharmacies must operate on two shifts, with a third shift if scheduled by the Ministry of Health [8].

Data shows that as of now, there are 1182 pharmacies in North Macedonia serving a population of 2,098,523, while in Bulgaria, there are 3258 pharmacies for a population of 6,747,168 [19,20,21,22].

Pharmacies in both countries are primarily concentrated in larger towns, with an uneven distribution across the territory, leading to challenges in pharmaceutical services in regions with smaller populations.

Table 2 summarizes and presents information on access to pharmacies relative to the population, the availability of 24 h pharmacies, and pharmaceutical services in smaller towns.

According to a study from 2019, the number of pharmacies in Bulgaria is 4.13 per 10,000 people, which is higher compared to the European average of 3.23 pharmacies per 10,000 people. In North Macedonia, the number is even higher, at 5.5 per 10,000 people. In North Macedonia, the number of pharmacies is greater, and between 2012 and 2019, their number has increased. According to statistical data, in 2019, one pharmacy served an average of 1800 people (5.5 pharmacies per 10,000 people), compared to 2012 when one pharmacy served 3000 people (3.3 pharmacies per 10,000 people) [23,24].

In Bulgaria, non-prescription medicines may be dispensed only in pharmacies or licensed drugstores. In contrast, in the Republic of North Macedonia, non-prescription medicinal products may be sold in grocery stores and petrol stations in settlements with more than 30,000 inhabitants—and, under specific conditions, even in those with populations of up to 30,000.

Pharmacies in settlements with more than 30,000 inhabitants must operate in two shifts, and according to a schedule set by the Ministry of Health, a third shift may also be required. A licensed pharmacist must be present in each shift. This requirement is practically difficult to fulfil due to the shortage of qualified pharmacists. A grace period for the implementation of this regulation was introduced, lasting from 2010 to 2020.

### 3.2. Policies for Ensuring Access to Pharmaceutical Services in Small Settlements

A comparison between the policies of the two countries for ensuring access to pharmaceutical services in small settlements is presented in Table 3. The table includes key aspects such as financial incentives, regulatory framework, staffing requirements, and other relevant factors.

In Bulgaria, the National Pharmacy Map identifies the regions, municipalities, and settlements with a shortage of open pharmacies by analyzing the population’s access to community pharmacies. The National Pharmacy Map was adopted by Decision No. 918 of 20 December 2023, by the Council of Ministers and was published in the State Gazette, issue No. 106 of 22, December 2023 [28,29]. Currently, the establishment of new community pharmacies is still not coordinated with it.

Macedonian legislation imposes restrictions on the establishment of pharmacies based on both demographic and geographic criteria. If a legal entity owns more than two pharmacies in a single settlement, or if it owns five pharmacies across the country, regardless of whether they are in the same municipality, it is required to open a pharmacy in a rural area for each of the five pharmacies. The Ministry of Health determines the location of pharmacies in rural areas based on an analysis of the population’s needs. If the legal entity fails to comply with the Ministry’s requirements, its pharmacies may be revoked, leaving it with only one pharmacy.

In Bulgaria, a Methodology for the Financing of Pharmacies has been established. According to this Methodology, the National Health Insurance Fund (NHIF) provides an additional amount/financial support to pharmacies that have a contractual agreement with the NHIF and meet specific criteria. Eligible pharmacies must operate for at least 40 h per week and fulfill at least one of the following conditions: be located in a remote area, be situated in a hard-to-reach settlement, be the sole provider of the contracted service within the municipality, or operate on a 24-h basis.

The Health Insurance Fund in the Republic of North Macedonia provides financial incentives for pharmacies in rural areas (located at least 10 km away from another pharmacy) that would not be able to survive financially under the existing payment method.

### 3.3. Educational System and Continuing Education:

Pharmacy education in Europe must comply with the higher education requirements established by the Bologna Declaration [30] and EU Directive 2005/36/EC [31]. The training of individuals in the field of pharmacy in both countries follows the established regulatory framework, but there are small differences. A comparison of the educational systems is presented in Table 4.

In Bulgaria, the specialty in the professional field of “Pharmacy” is regulated by law. According to the Higher Education Act and Directive 2005/36/EC, as amended by Directive 2013/55/EU, a “regulated profession” is defined as an activity or a set of activities that are included in the List of Regulated Professions in the Republic of Bulgaria. These professions are considered to be of public significance and/or essential for the life and health of people. The right to practice them is granted based on legal, secondary legal (sublegal), or administrative regulations [30,31,32]. In Bulgaria, the provisions of Directive 2005/36/EC are implemented through the Professional Qualifications Recognition Act (PQRA) [33]. A regulation has defined the unified state requirements for obtaining higher education in the specialty “Pharmacy” for the educational qualification degree “Master” [34].

In Bulgaria, higher education in the specialty “Pharmacy” is acquired in a faculty of an accredited higher education institution in accordance with the Higher Education Act. The studies are full time with a duration of no less than 5 academic years, including theoretical training lasting no less than nine semesters and practical training—educational internships (Botany and Pharmacognosy) and a 6-month pre-graduation internship after the 9th semester. It concludes with state exams conducted by a state examination commission, comprising three qualified lecturers in the relevant core discipline from the higher education institution [35].

In North Macedonia, pharmacy specialists are either Master’s degree pharmacists—specialists with a higher education who have earned 300 or 360 ECTS (European Credit Transfer and Accumulation System) credits, or technicians with 180 ECTS credits [36]. In North Macedonia, the duration of the Master’s degree in Pharmacy is 5 years, which includes one year of internship. After completing the program, each graduate pharmacist must take an exam to obtain a license from the Chamber of Pharmacists. As part of the program approved by the Ministry of Health, the graduates appear in the Chamber and complete a six-month internship in health institutions under the supervision of authorized educators who meet the established requirements. After obtaining the signatures in a special intern booklet, they take an exam to obtain a license before a commission formed by the Chamber of Pharmacists. The members of the commission must meet the criteria set by the Regulation approved by the Ministry of Health. Technicians receive their education in specialized secondary schools.

### 3.4. Responsibilities of Staff in Pharmacies

Pharmacies employ individuals who have acquired the appropriate educational qualification level (EQL) according to the legislation. In pharmacies in Bulgaria, only Master’s degree pharmacists and pharmacy assistants are allowed to work, whereas in North Macedonia, both Master’s degree pharmacists and pharmaceutical technicians are employed. A comparison of the rights and responsibilities between professionals in both countries is presented in Table 5.

In both countries, the operations in the pharmacy are carried out by a master pharmacist according to the current regulatory provisions, with the pharmacy assistant/technician being allowed to perform all activities in the pharmacy under the supervision of a master pharmacist, except for: dispensing medication according to a doctor’s prescription, supervising, and providing consultations related to medicinal products [5,8,11,13].

To qualify as a pharmacy manager, a master pharmacist must have at least one year of professional experience in a pharmacy and must not have a criminal record. The pharmacy manager is responsible for the overall organization of the pharmacy’s workflow in accordance with the applicable legislation [11].

### 3.5. Maintaining Professional Competencies of Pharmacists

Pharmacists are required to maintain their professional competencies to ensure the provision of adequate pharmaceutical care. The structure and regulation of continuing education differ between the two countries.

In Bulgaria, according to the Health Act, the professional organizations of master pharmacists organize, coordinate, conduct, register, and oversee the continuing medical education of pharmacists in accordance with agreements with higher education institutions, the Bulgarian Red Cross, and the Military Medical Academy [37]. Every master pharmacist, who is a regular member of the Bulgarian Pharmaceutical Union, must complete forms of continuing education within one calendar year, accumulating a total of no less than 30 credit points [38,39]. In the Republic of North Macedonia, the initial work license is renewed every seven years through participation in continuing education courses accredited by the Chamber of Pharmacists [40,41].

## 4. Discussion

In Bulgaria, the activities related to medical devices are regulated by a separate act, and additional provisions are specified in accompanying regulations [42]. The increased supervision over the safety of medical devices is also included in the National Health Strategy 2030 [43]. At the European Union level, the regulatory framework governing medical devices and in vitro diagnostic medical devices is established by Regulation (EU) 2017/745 on medical devices and Regulation (EU) 2017/746 on in vitro diagnostic medical devices. As a Member State, Bulgaria is required to align its national legislation and regulatory practices with the provisions set out in these binding EU regulations [44,45]. In contrast, in North Macedonia, there is no standalone law specifically regulating medical devices; however, the sector is governed through various secondary legislative instruments, such as rulebooks, guidelines, and administrative instructions, which collectively aim to provide a basic regulatory framework, albeit less comprehensively and systematically.

In Bulgaria, the requirements for the organization of work and the nomenclature in pharmacies and drugstores are clearly outlined in separate regulations from the MPHMA [11], p. 28, [12]. Similarly, legislation in North Macedonia follows a similar approach. The activities of pharmacies are regulated by two laws—the Law on Medicines and Medical Devices and the Law on Health Care—as well as by secondary legislation, including the Rules on Premises, Equipment, and Professional Staff for Establishing, Commencing Operations, and Performing Health Activities in Healthcare Institutions.

In 2019, the World Health Organization (WHO) issued a statement emphasizing that pharmacists must be involved in meeting the growing healthcare needs of society. The population should benefit from universal and appropriate access to pharmacies for public service through measures such as allowing for the planning of the number of pharmacies, which can be achieved through licensing based on demographic and/or geographical criteria and/or economic incentives; and optimizing and expanding the scope of existing pharmacies (for example, by creating pharmacy branches) [46].

In many studies, territorial accessibility is determined through the density of the pharmacy network and/or the distance that patients must travel to reach a pharmacy [47]. In the USA, in 2022, the nearest pharmacies were located 2 miles away from patients [48]. In Portugal, 80% of the adult population lives within a 10 min walk to a pharmacy. In Nova Scotia, Canada, the distance to pharmacies in relation to the population is as follows: 42% live within 800 m, 62.6% within 2 km, and 78.8% within 5 km. In urban areas, 61.3% live within 800 m, 90% within 2 km, and 99.2% within 5 km; in rural areas, 28% live within 2 km, and 53.3% live within 5 km [49].

Studies have shown that the lowest pharmacy density is reported in South Africa—0.61 pharmacies per 10,000 people—while one of the highest is in Canada, with 12.24 pharmacies per 10,000 people. Based on this, it can be concluded that both Bulgaria (4.13/10,000) and North Macedonia (5.5/10,000) are at an average level in terms of pharmacy accessibility per 10,000 people globally. In both countries, with the shift to private ownership, the number of pharmacies serving the population has increased, and their number is slightly above the European average of 3.3 pharmacies per 10,000 people [50].

In both North Macedonia and Bulgaria, pharmacies are unevenly distributed by regions. North Macedonia offers a better round-the-clock service and physical accessibility in smaller towns through 24/7 duty pharmacies operating on a specific schedule, as well as а legislative draft for the establishment of mobile pharmacies, including those designated to serve remote settlements with populations not exceeding 1000 inhabitants. Access is further supported by regulatory measures, such as restrictions on the number of pharmacies that a single owner can operate in larger cities and mandatory pharmacy openings in small towns. The imposition of stringent sanctions for non-compliance with this statutory provision compels pharmacy owners to establish outlets in rural areas, thereby enhancing access to pharmaceutical services. The determination of these locations falls under the authority of the Minister of Health [17].

In contrast, Bulgaria is exploring alternative mechanisms to improve access in underserved areas, including financial support for pharmacies in small towns and the implementation of the National Pharmacy Map [28].

In Europe, the average number of full-time pharmacists is three per pharmacy. In Bulgaria, however, the average is 2.1, with some regions, such as Pazardzhik, reporting an average as low as 0.9 [24].

There is a noticeable concentration of specialists in larger cities and a shortage of pharmacists in small settlements [51]. Studies in Australia highlight that pharmacists in small towns face challenges such as a lack of financial incentives, and professional and social isolation. The key factor influencing their decision to work in these areas is the opportunity to collaborate within a multidisciplinary team [47]. Similarly, previous studies in Bulgaria indicate that pharmacists are willing to work in small towns if offered motivating salaries [52]. Consequently, legislative changes and financial support aimed at opening and operating pharmacies in small settlements [52] (p. 32) are being introduced to address this issue and improve access to specialists in these areas.

In Bulgaria, pharmacy assistants have a higher level of education compared to technicians in North Macedonia, which contributes to a higher level of competence. However, this advanced education does not result in expanded responsibilities within the pharmacy setting. In Bulgaria, master pharmacists complete their education through semesters of coursework, mandatory practical training, and by passing a state exam administered by a commission of three qualified academic staff members from higher medical schools. In contrast, in North Macedonia, the primary responsibility for acquiring professional qualifications lies with the professional association, specifically the Chamber of Pharmacists [53].

According to the FIP, in alignment with the Development Goals (DGs), every pharmacist is required to further develop their professional career through post-graduate education and skill development after completing their university studies [26,54].

The FIP provides guidelines for continuing education, emphasizing the need for regulation through legislation and the introduction of rules by professional organizations [55]. A comparison between the two countries reveals that both have established clear rules for conducting continuing education to maintain professional competence. The responsibility for organizing and implementing continuing education lies with the professional organizations.

In 2022, the number of practicing pharmacists in North Macedonia was significantly lower, at 5.95 per 10,000 inhabitants, compared to 8.91 per 10,000 in Bulgaria. According to Eurostat data, the number of pharmacy graduates in 2021 was notably higher than in 2011. In both countries, the number of graduates was almost identical in 2011 and 2021, with North Macedonia graduating 2.7 pharmacists per 10,000 inhabitants in 2011 and 5.9 in 2021, while Bulgaria graduated 2.4 in 2011 and 5.68 in 2021 [56,57,58].

The majority of other EU countries reported between 5.8 and 12.3 pharmacists per 10,000 inhabitants, with the lowest rate recorded in the Netherlands at 2.17 per 10,000 in 2022. The increased number of qualified specialists could also positively impact access to pharmaceutical services in small settlements by ensuring the availability of personnel in these areas. Bulgaria and North Macedonia share a post-socialist history that shaped the liberalization of their pharmaceutical sectors. In both countries, community pharmacies are currently under private ownership, with national medicines agencies (BDA and MALMED) overseeing their authorization and supervision. This comparative study highlights both similarities and differences in the pharmaceutical legislation and organization of community pharmacy services in Bulgaria and North Macedonia, providing insights into how regulatory frameworks affect access to pharmaceutical care.

### Limitations of the Study

The limitations of the study are related to the lack of statistical data for comparative analysis over recent years. Additionally, inconsistencies in data presentation across different sources pose a challenge to the accuracy and reliability of the findings.

## 5. Conclusions

Both countries face similar challenges in regulating the retail trade of medicinal products, particularly in ensuring access to pharmaceutical services in small settlements and encouraging the establishment of pharmacies in these areas.

North Macedonia demonstrates improved accessibility to pharmaceutical services, attributable to measures such as the provision of services in small settlements, the introduction of mandatory obligations for pharmacy chains to establish outlets in rural areas, and the implementation of duty schedules ensuring continuous, 24 h pharmaceutical coverage.

In contrast, Bulgaria is introducing financial incentives to ensure the availability of pharmaceutical services in smaller settlements. Furthermore, North Macedonia has a more robust control system for continuing education, suggesting that the competence of practicing pharmacists is actively monitored and maintained. In both countries, continuing education is organized by professional organizations.

Each country is exploring different mechanisms to address the challenges of accessing pharmaceutical services. The exchange of experiences between the two countries could be beneficial for the further development of the pharmaceutical sector.

## Figures and Tables

**Table 1:** Comparative overview of the legal requirements related to ownership and types of pharmacies in Bulgaria and North Macedonia.

Criteria	Bulgaria	North Macedonia
**Types of Pharmacies**		
Community pharmacies	Yes	Yes
Hospital pharmacies	Yes	Yes
Pharmacy point unit of a main pharmacy, typically located in underserved or remote areas	No	Yes
Mobile pharmacies	No	Yes
Limitations regarding the spatial separation between two pharmacies	No	Yes/a minimum distance of 100 m is required between pharmacies in settlements with populations of up to 4500 residents
**Ownership**		
Natural or legal person	Yes	Yes
**Restrictions on Opening Pharmacies**		
Horizontal integration	A single owner is allowed to operate up to four pharmacies.	There are no limitations on the number of pharmacies that may be owned by a single individual or entity.
Vertical integration	Regulated by legislation (not permitted)	Regulated by legislation
Requirements for the holder of the retail pharmacy license (The responsible pharmacist/master pharmacist)	Minimum of 1 year of professional experience;	A master pharmacist may open a pharmacy if there is no conflict of interest with positions in the Ministry of Health or the National Medicines Agency.
	The responsible pharmacist (master pharmacist) may own a retail license only for one pharmacy and must work at that pharmacy;	
	A person who has obtained a retail permission for medicinal products in a pharmacy cannot be the owner or participate in commercial companies, or be employed in another commercial company engaged in the production, import, wholesale, or retail trade of medicinal products	

**Table 2:** Access to pharmacies and pharmaceutical services in Bulgaria and North Macedonia.

Indicator	Bulgaria	North Macedonia
Number of pharmacies per 10,000 inhabitants as of 2019	4.13	5.5
Provision of 24 h pharmacies	Problem in some regions	List of on-call pharmacies
Provision of pharmaceutical services in small settlements	Problem in small settlements	Mobile pharmacies are planned for settlements with up to 1000 inhabitants

**Table 3:** Comparison of mechanisms for ensuring access to pharmacies in small settlements in Bulgaria and North Macedonia [25,26,27].

Category	Bulgaria	North Macedonia
Regulatory Requirements	There are no regulatory restrictions on the number of pharmacies, as the policy aims to promote the establishment of pharmaceutical facilities in smaller and underserved settlements.	Owners of more than two pharmacies in one settlement or more than five pharmacies in the country are required to open a pharmacy in a rural area.
Needs Assessment for Pharmacies	National pharmacy map	The Ministry of Health determines the location of pharmacies in rural areas
Staff Requirements	In the absence of pharmacy in the area: ✓ A master pharmacist can be a license holder without the mandatory one-year experience if there is no other pharmacy. ✓ The assistant pharmacist can be a retail permit owner if there is no other pharmacy in the locality and the pharmacy dispenses only over-the-counter medicines.	Pharmacist required; more flexible service schemes are being considered (e.g., mobile pharmacies or duty shifts).
Availability of Mobile Pharmacies	Not legislatively regulated or allowed.	A project approved for the possibility of establishing mobile pharmacy points in settlements with fewer than 1000 inhabitants.
Health Insurance Funds	Pharmacies dispensing medicines paid by the National Health Insurance Fund (NHIF) may receive financial support if they meet the criteria in an approved payment scheme.	Pharmacies can contract with the Health Insurance Fund (“Fund for Health Insurance”); more flexible policies in some regions.
Other Incentives	Opportunities through European projects for rural areas (e.g., Rural Development Programme). Funding from regional programs.	National healthcare programs focused on rural areas.

**Table 4:** Comparison of educational systems.

Characteristics/Criteria	Bulgaria	North Macedonia
Master pharmacist		
Education	Higher education	Higher education
Duration of education	5 years	5 years, including one year of internship
Pre-graduation internship	6 months	6 months
Qualification	State exams, before a state commission of accredited academic staff	Exam for obtaining a license before the Chamber of Pharmacists
Mandatory membership in a professional organization	Yes	Yes
**Pharmacy assistants/technicians**	**Pharmacy assistants**	**Technicians**
Education	Medical college	Secondary education

**Table 5:** Comparison of the responsibilities of master pharmacists and pharmacy assistants/technicians in Bulgaria and North Macedonia.

Responsibilities		
Specialist	Bulgaria	North Macedonia
Master pharmacist	All operations in the pharmacy	All operations in the pharmacy
Pharmacy assistants/technicians	All operations in the pharmacy under the supervision of a master pharmacist, except for the dispensing of prescription medications	All operations in the pharmacy under the supervision of a master pharmacist, except for the dispensing of prescription medications, including in mobile pharmacies and pharmacy points, where the master pharmacist must carry out control at least once a week

## Data Availability

The instruments and the dataset used and analyzed during the current study are available from the corresponding author on request.

## Abbreviations

The following abbreviations are used in this manuscript:

BDA	Bulgarian Drug Agency
BPhU	Bulgarian Pharmaceutical Union
DGs	Development Goals
EDQM	The European Directorate for the Quality of Medicines and Healthcare
EMA	European Medicines Agency
EQL	Educational qualification level
EU	European Union
FIP	International Pharmaceutical Federation
GPP	Good Pharmacy Practice
HIF	Health Insurance Fund
MPHMA	Medicinal Products in Human Medicine Act
NHIF	National Health Insurance Fund
PQRA	Professional Qualifications Recognition Act
WHO	World Health Organization
